# Association between Obesity, Overweight, Elevated Waist Circumference, and Insulin Resistance Markers among Brazilian Adolescent Students

**DOI:** 10.3390/nu14173487

**Published:** 2022-08-24

**Authors:** Rodolfo Deusdará, Amanda de Moura Souza, Moyses Szklo

**Affiliations:** 1 Faculdade de Medicina, Universidade de Brasília, UnB, Campus Universitário Darcy Ribeiro, Asa Norte, Brasilia 70910-900, DF, Brazil; 2 Instituto de Estudos em Saúde Coletiva, Universidade Federal do Rio de Janeiro, Avenida Horacio Macedo, S/N-Próxima à Prefeitura Universitária da UFRJ, Ilha do Fundão-Cidade Universitária, Rio de Janeiro 21941-598, RJ, Brazil; 3 Epidemiology Department, Johns Hopkins Bloomberg School of Public Health, 615 N. Wolfe Street Room W6009, Baltimore, MD 21205, USA

**Keywords:** insulin resistance, biomarkers, adolescent, obesity

## Abstract

(1) Background: There is still controversy concerning the most effective and efficient strategy to identify insulin resistance in adolescents. We estimated the level of fasting insulin (fasting insulin equivalent, FIeq) that would replicate the strength of the associations of obesity, overweight, and waist circumference with two insulin resistance markers: triglyceride/high-density lipoprotein (TG/HDL) and triglyceride/glucose (TyG); (2) Methods: We studied approximately 38,000 adolescents aged 12 to 17 years, sampled from a multicenter Brazilian school-based survey, The Study of Cardiovascular Risk Factors in Adolescents (Portuguese acronym, ERICA), conducted in 2013–2014. Fasting insulin equivalents for adiposity variables were calculated by dividing the beta coefficient of each adiposity measure by the fasting insulin beta coefficient from linear regression analysis according to age (12–14, 15–17 years old) and sex, and adjusted by smoking, alcohol consumption, physical inactivity, sedentary behavior, socioeconomic status, and Tanner stage; (3) Results: The FIeqs for obesity were greater than those for overweight and elevated waist circumference for both TG/HDL and TyG in early adolescence. The FIeqs for elevated WC were greater than those for obesity and overweight in adolescents aged 15 to 17 years; (4) Conclusions: Our study suggests that WC measurements might be useful to identify adolescents with insulin resistance, particularly in late adolescence.

## 1. Introduction

Insulin resistance in adolescents is characterized by a reduced insulin sensitivity, followed by compensatory hyperinsulinemia in order to maintain an euglycemic state [1], and it increases the likelihood of some conditions such as glucose intolerance, dyslipidemia, endothelial dysfunction, procoagulant factors, hemodynamic changes, markers of inflammation, increased testosterone secretion, and sleep-disordered breathing [2]. Thus, there is an increasing interest in the early identification of adolescents with insulin resistance in clinical routine practice [3,4]. Clinical guidelines recommend the measurement of body mass index (BMI, weight in kilograms divided by the square of height in meters) to detect obesity and overweight in primary care settings [5]. However, previous studies have found conflicting results about the best strategy to identify insulin resistance in adolescents, that is, BMI alone, waist circumference (WC) alone, or BMI and WC [3,6,7].

The direct method for measuring insulin sensitivity, regarded as the gold standard, is the hyperinsulinemic–euglycemic clamp [8]; however, this procedure is time-consuming, expensive, invasive, and labor-intensive [8]. Indirect methods of measuring insulin resistance are typically based on insulin and/or glucose levels, such as the homeostasis model assessment insulin resistance (HOMA-IR). Other insulin resistance markers have also gained popularity [9], including the triglyceride/high-density lipoprotein (TG/HDL) and triglyceride/glucose (TyG) index [9]. Correlation coefficients between these markers and the hyperinsulinemic–euglycemic clamp in adolescents have been shown to be r = 0.82 for HOMA-IR, r = 0.695 for TyG index, and r = 0.416 for TG/HDL [9,10].

These insulin resistance markers are related to different aspects of glucose homeostasis and express different components of insulin resistance [11]. They also have different analytic units, which makes it difficult to compare the strength of their associations with adiposity variables. Using an LDL equivalent (based on the fact that lipids are a necessary component of atherosclerosis), Sharrett AR, et al. (2004, 2006) calculated the concentration of LDL (which he named “LDL equivalent”) that would replicate the strengths of the associations of smoking and diabetes with different phases of the natural history of atherosclerosis [12,13]. Using a similar approach, we compared obesity, overweight, and elevated WC with two insulin resistance markers. More specifically, we estimated the level of fasting insulin (fasting insulin equivalent, FIeq) that would replicate the strength of the associations of the obesity, overweight, and elevated WC with TG/HDL and TyG. We considered fasting serum insulin levels to be a reasonable “Sharrett equivalent” (i.e., the basis for comparison), as compensatory hyperinsulinemia is the defining characteristic of insulin resistance. Thus, the aim of our study was to analyze the association between fasting insulin equivalents for adiposity variables (obesity, overweight, and elevated waist circumference) and insulin resistance markers in Brazilian adolescents.

## 2. Materials and Methods

### 2.1. Study Design and Sample

ERICA is the Portuguese acronym for The Study of Cardiovascular Risk Factors in Adolescents, a multicenter Brazilian school-based cross-sectional survey of 75,000 adolescents aged 12 to 17 years from 1247 schools from 122 municipalities with more than 100,000 inhabitants, conducted in 2013–2014. The study’s multistage sampling used stratification into 32 geographical areas (27 State capitals and 5 macro-regions) [14,15]. The sample of 75,000 reflected the exclusion of 4 schools that refused participation, located in 2 municipalities [16]. Among the 72,508 students, a subsample of 37,815 (52.2%) morning shift adolescents from 111 municipalities had complete information on questionnaire data, anthropometrics, and blood pressure. This subsample had their fasting blood samples collected [16]. 

The study was approved by local Ethics Committees [15]. More details of the study design have been published [14,15].

### 2.2. Anthropometric Measures

Adolescents wore light clothes and no shoes during the anthropometric measurements. Height and weight were measured by a portable calibrated stadiometer, Alturexata^®^, and by a digital scale, model P150m, Líder^®^, respectively [15]. The nutritional status categories were defined based on age- and sex-specific BMI levels. Obesity, overweight, and normal nutritional status were defined as follows: Z-score > +2; Z-score > +1 and Z-score ≤ +2; Z-score ≥ −2 and Z-score ≤ +1, respectively [17].

WC was measured using fiberglass anthropometric tape, Sanny^®^. As suggested by the World Health Organization, the measurement was done at the medium point between the lower costal margin and the highest point of iliac crest [15]. Values ≥ 90th percentile for those aged 10 to <16 years old, and ≥90 cm for males and ≥80 cm for females for those aged 16 years or older were defined as elevated WC [18].

### 2.3. Biochemical Assays

Only morning shift students had their bloods collected [15] and, as mentioned previously, constitute our analytic sample. All students were instructed to fast for 12 h the night before, and blood was collected the following morning. A detailed description of blood collection procedures as well as quantitative internal and external quality control procedures have been published [19]. Serum glucose, insulin, HDL-cholesterol, and triglyceride levels were measured using the hexoquinase, chemiluminescence, enzymatic colorimetric, and enzymatic kinetic assays, respectively.

### 2.4. Outcome Definitions

Insulin resistance markers’ definitions were based on the following variables and equations [9,20,21]:

TG/HDL: [fasting TG (mg/dL) × 0.0113]/[HDL-c (mg/dL) × 0.0259]

TyG: Ln [fasting TG (mg/dL) × fasting glucose (mg/dL)/2]

### 2.5. Statistical Analysis

ERICA’s complex sampling design and sampling weights were considered in the analyses [14].

Absolute and relative frequencies were calculated for categorical variables. For continuous variables, normality was evaluated by the Shapiro–Wilk test. No variable had a normal distribution. Thus, medians and interquartile ranges were calculated for continuous variables.

Associations of adiposity variables with insulin resistance markers were evaluated using a multivariable regression linear model. We calculated coefficients of each independent variable stratified by age and sex. Covariates included physical inactivity (<420 min of physical activity per week), smoking status (≥1 cigarette smoked at least one day in the last 30 days), sedentary behavior (≥3 h a day spent with television, video games, or computer in an ordinary weekday), alcohol consumption (≥1 alcoholic drink on at least one day in the last 30 days), socioeconomic status (defined by whether the adolescent attended public or private schools), and Tanner stage.

Calculation of the fasting insulin equivalent (FIeq) was based on the method proposed by Sharrett AR, et al. [13]; the FIeq represented the level of fasting insulin that would replicate the strength of the association of each adiposity variable with each insulin resistance marker. In our study, following Sharrett’s suggested procedure [12,13], the FIeq was calculated by dividing the beta coefficient of each adiposity measure by the fasting insulin beta coefficient from linear regression analysis. For example, the FIeq for elevated waist circumference was calculated by dividing the beta coefficient of elevated waist circumference by the beta coefficient of fasting insulin from the linear regression analysis.

For example, in boys between 15 and 17 years old and for the outcome TG/HDL, the coefficient for the association of insulin was 0.02, which estimates the independent association of a 1 mU/L increase of fasting insulin with the value of TG/HDL. The coefficient for elevated WC was 0.58, and division by the insulin coefficient of 0.02 shows that the elevated WC association with TG/HDL was equivalent to a fasting insulin value of 29 mU/L. This value, 29 mU/L, is our estimated fasting insulin equivalent (FIeq) for WC pertaining to TG/HDL

We decided not to use HOMA-IR as one of the markers of insulin resistance, as insulin was used in the model as the “equivalent” variable and is also included in the HOMA-IR equation.

All FIeq estimates and respective confidence intervals were calculated using the jackknife method (for the presentation below, all regression (beta) coefficients were rounded to two decimals places).

Due the different cut-off values of insulin resistance markers and based on the linear regression results, we conducted a secondary analysis with Poisson regression models. We used the 75th percentile value as the cut-off value of TyG and TG/HDL.

## 3. Results

### 3.1. Description of the Study Population

The median age of our study sample was 15 years. Almost 30% of all adolescents were classified as having excess weight (overweight + obesity), with obesity comprising almost 9% (Table 1). Approximately 22% of adolescents consumed at least 1 alcoholic drink in the last 30 days, and less than 5% smoked at least one cigarette in the last 30 days. Approximately 40% of adolescents had sedentary behavior. Most adolescents were in the pubertal stage (Tanner stages 2, 3, and 4), physically inactive, enrolled in public schools, and had a normal WC. Of the markers of insulin resistance, TyG showed the least variability.

### 3.2. Comparison of Association Strengths of Adiposity Variables within Each Insulin Resistance Marker (TyG and TG/HDL)

#### 3.2.1. TyG

In adolescents aged 12 to 14 years, regression (beta) coefficients for obesity were greater than those for both overweight and elevated WC, particularly in girls (Table 2). However, the association of elevated WC was significantly stronger than the associations for obesity and overweight in adolescents aged 15 to 17 years, particularly in boys.

#### 3.2.2. TG/HDL

Elevated WC was more strongly associated with TG/HDL than obesity and overweight were in adolescents aged 15 to 17 years. For example, in boys 15 to 17 years old, elevated WC and overweight were greater by 0.58 and 0.03 TG/HDL units, compared with normal WC and nutritional status, respectively (Table 2). However, in adolescents aged 12 to 14 years, the coefficient for obesity was significantly greater than the coefficients for overweight and elevated WC, particularly for boys.

### 3.3. Comparison between Insulin Resistance Markers Using FIeq

#### 3.3.1. Obesity

**Figure 1:**
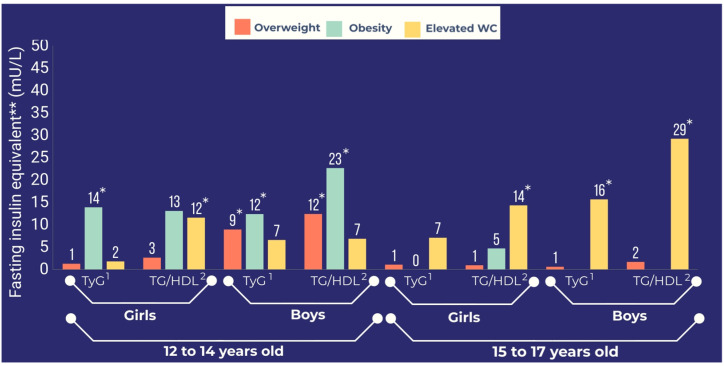
Adjusted ** fasting insulin equivalents for overweight, obesity, and elevated waist circumference for insulin resistance markers according to age and sex in The Study of Cardiovascular Risk Factors (2013–2014). ** Adjusted for physical inactivity, smoking status, sedentary behavior, alcohol consumption, socioeconomic status, and Tanner stage; ^1^ triglyceride/glucose index; ^2^ triglyceride/high-density lipoprotein; * *p*-value ≤ 0.05.

The FIeq for obesity was greater than the FIeqs for overweight and elevated WC for TyG and TG/HDL in adolescents aged 12 to 14 years, particularly in boys (Table 3; Figure 1). However, in adolescents aged 15 to 17 years, the FIeq for obesity tended toward to the null value for TyG and TG/HDL, with the exception of TG/HDL for girls (Table 3; Figure 1).

#### 3.3.2. Overweight

The FIeq for overweight tended toward the null for TyG and TG/HDL, except for boys aged 12 to 14 years (Figure 1).

#### 3.3.3. Elevated WC

As for TyG and TG/HDL, the FIeq for elevated WC was greater in late adolescence than in early adolescence in boys and girls (Figure 1).

In adolescents aged 15 to 17 years, the FIeq for elevated WC was greater than the FIeqs for obesity and overweight for TG/HDL and TyG (Table 3). For example, the adjusted FIeqs for elevated WC were approximately 29 mU/L and 6.9 mU/L for TG/HDL in boys aged 15 to 17 years and 12 to 14 years, respectively (Table 3).

### 3.4. Secondary Analysis with Poisson Regression and the 75th Percentile Value as the Cut-Off Value for Two Insulin Resistance Markers (TyG and TG/HDL)

The FIeq for obesity vs. TyG and TG/HDL was greater than those for overweight and waist circumference in early adolescence, except for TyG and TG/HDL in late adolescence (Supplementary Figure S1).

## 4. Discussion

In our study, obesity was shown to have stronger associations with TG/HDL and TyG in adolescents aged 12 to 14 years than in older adolescents. However, in adolescents aged 15 to 17 years, WC showed stronger associations with TG/HDL and TyG than obesity did.

In a prospective study, which did not use equivalents, of 5232 children aged 9–12 years, BMI alone was a better predictor of cardiovascular risk factors than WC was [22]. However, in another study with adolescents, WC explained a greater variance in abdominal obesity and insulin sensitivity than BMI percentile did [6].

Adiposity accounts for more than one-half of the variability in insulin sensitivity in normal children [23]. BMI and WC are strong surrogate measures of adiposity [24]. BMI does not distinguish between fat and muscle or bone, or consider age-related changes in body composition [25]. However, BMI has been shown to be strongly associated with X-ray absorptiometry (DXA)-determined total body fat and percent body fat in children and adolescents [26]; although, the correlation seems to decrease in older ages [27]. WC has been found to be highly correlated with DXA fat trunk [28]. On the other hand, WC cannot satisfactorily distinguish between visceral and subcutaneous fat [29].

As a consequence of the increased release of free fatty acid (FFA) from adipose tissue to non-adipose tissue in an insulin resistance state, there is fatty acid accumulation in non-adipose tissues, such as liver, muscle, and heart, and hypertriglyceridemia [30]. Ectopic fat deposition with lipotoxicity has been recognized as underlying insulin resistance [30]. TG level has been shown to be negatively correlated with insulin sensitivity [31] and positively correlated with the progression of insulin secretion dysfunction [31]. Childhood obesity is related to the atherogenic lipid profile [31]. TyG index, which is a product of triglyceride and fasting glucose, was found in a previous study to be correlated with adiposity, metabolic parameters, and markers of subclinical atherosclerosis related to insulin resistance [9].

In adolescents with obesity, the TyG index was superior to TG/HDL and inferior to fasting insulin with the hyperinsulinemic–euglycemic clamp [9]. In a Brazilian study of adults, TyG performed slightly better than HOMA2-IR regarding the hyperglicemic clamp, as the outcome [32].

Visceral abdominal fat in adults is associated with insulin resistance [33]. In adults, an elevated WC is a better marker of metabolic risk than is an elevated BMI [34]. WC has been found to be more strongly correlated with TG/HDL than BMI is in adults [35]. In adults with newly diagnosed type 2 diabetes mellitus, BMI and WC were weakly correlated with TG/HDL [36]. WC appears to have a stronger correlation with TyG than BMI does in adults [37]. However, previous studies have shown conflicting results in children and adolescents [6,22]. In Malaysian children with obesity and overweight aged 9 through 16 years [38] and Argentinian adolescents [39], TG/HDL was found to be more strongly correlated with WC than with BMI. However, in a UK longitudinal cohort of 5000 children aged 9–12 years, BMI was associated with cardiovascular risk factors at comparable magnitudes of association to those of fat mass and waist circumference [22].

Within our analysis using fasting insulin equivalents, TG/HDL was more strongly associated with adiposity variables than TyG was. One possible explanation is that visceral obesity contributes to insulin resistance and is also related to the dyslipidemic profile. The increased flux of free fatty acids from adipose tissue to the liver augments hepatic triglyceride synthesis. The hepatic lipid accumulation appears to cause hepatic insulin resistance. Moreover, a low HDL cholesterol level, due to hypercatabolism of HDL in the insulin-resistant state, is more common in patients with insulin resistance than hypertriglyceridemia is [40].

Increases in body fatness, particularly adipose tissue in the abdomen of children and adolescents, is associated with abnormal lipids and insulin profiles [31,40]. In a previous study, WC alone was consistently associated with lipid concentrations in adolescents [41]. In young adults, WC, and not BMI, has shown a strong association with TG and TC/HDL cholesterol [42]. In adults, triglycerides levels are more strongly correlated with WC than with BMI. Previous studies have reported that there is an increase in serum TG concentrations from early to late adolescence [43]. In addition, there is a reduction in HDL-C concentrations, as well as an increase in TG concentrations throughout pubertal development [43]. In puberty, females have gynecoid body distribution, especially on the hips and thighs. However, males have an android body shape with excessive accumulation of abdominal fat, and relatively stable peripheral fat [44]. Adolescent boys may have more visceral abdominal fat than total abdominal adipose tissue [44,45]. Based on the fat distribution pattern, girls have more fat in the hip and less in the waist compared to boys [44]. In adults, visceral adipose tissue was more strongly associated with cardiometabolic risk factors than subcutaneous adipose tissue was, independently of BMI [46]. Moreover, WC is a better predictor of visceral adipose tissue compared to BMI in children and adolescents [6]. Thus, WC measurement may be more indicative of insulin resistance in boys than in girls [45]. Our study suggests that WC might be more appropriate than BMI for identifying insulin resistance in late adolescence when considering specific insulin resistance markers that do not consider insulin, such as TG/HDL and TyG.

### Limitations

Our study was cross-sectional and, thus, subjected to selection/survival and temporal biases. However, survival bias has likely not occurred, given the young ages of our study sample. On the other hand, temporal bias may have occurred, notwithstanding the biological plausibility of our findings. In addition, despite the multivariable adjustment, as for all single observational studies, residual confounding may have occurred.

The major strengths of ERICA are its school-based, multicenter, nationwide design, rigorous quality assurance and control, and the comparison between adiposity variables and insulin resistance markers using FIeq. The study results add to previous studies that lacked a strategy for comparisons of association strengths between adiposity and insulin resistance markers.

## 5. Conclusions

Our study suggests that WC measurements could be useful to identify adolescents with insulin resistance, particularly in late adolescence. Future studies should use a prospective design and include additional insulin resistance markers, such as TyG and TG/HDL. These markers could be valuable in the early identification of insulin resistance in primary care settings.

## Figures and Tables

**Table 1:** Characteristics of 37,815 adolescents enrolled in the Study of Cardiovascular Risk Factors in Adolescents (ERICA, 2013–2014).

Variables	n			
Continuous		Median	1°Q	3°Q
Age (years)	37,815	15	13	16
TG/HDL ^δ^	37,706	0.65	0.47	0.91
TyG ^Φ^	37,559	8.0	7.73	8.28
Insulin (mU/L)	37,760	8.3	5.8	11.7
Categorical		(%)	95% CI	
Female	22,682	50.2	-	-
Smoking (≥1 cigarette smoked in the last 30 days)	1406	4.2	3.8	4.7
Alcohol consumption (≥1 drink in the last 30 days)	7685	21.6	20.3	23.0
Sedentary behavior ^γ^	14,133	40.5	38.9	42.1
Physical inactivity (<420 min per week)	24,713	62.7	61.7	63.8
Public Schools	27,990	77.8	72.4	82.3
Tanner Stage				
Stage 1	172	0.5	0.4	0.6
Stage 2	1917	5.6	4.9	6.2
Stage 3	6651	16.9	16.0	17.9
Stage 4	14,889	40.0	38.5	41.5
Stage 5	14,162	37.0	35.7	38.3
Nutritional status				
Normal ^a^	27,073	71.0	69.4	72.5
Overweight ^b^	6635	17.5	44.0	49.2
Obesity ^c^	3097	9.2	8.5	10.0
Waist circumference (cm)				
Normal	33,373	87.4	86.3	88.4
Elevated ^d^	4386	12.6	11.6	13.7

^δ^ Triglyceride/high-density lipoprotein; ^Φ^ triglyceride/glucose index; ^γ^ ≥3 h a day spent with television, video games, or computer in an ordinary weekday; ^a^ Z-score ≥ −2 and Z-score ≤ +1; ^b^ Z-score > +1 and Z-score ≤ +2; ^c^ Z-score > +2; ^d^ ≥90th percentile for those aged 10 to <16 years old; ≥90 cm for males and ≥80 cm for females for those aged 16 years or older.

**Table 2:** Unadjusted and multivariable adjusted ^‡^ linear regression coefficients (absolute differences) and 95% confidence intervals expressing associations of waist circumference, overweight, and obesity with insulin resistance markers stratified by sex and age in 37,815 adolescents included in the Study of Cardiovascular Risk Factors (ERICA, 2013–2014).

Girls								
	12–14 years				15–17 years			
	TyG ^Φ^		TG/HDL ^δ^		TyG ^Φ^		TG/HDL ^δ^	
	Unadjusted	Adjusted	Unadjusted	Adjusted	Unadjusted	Adjusted	Unadjusted	Adjusted
Overweight ^b,f^	0.01 (−0.04, 0.05)	0.02 (−0.02, 0.06)	0.03 (−0.02, 0.09)	0.04 (−0.02, 0.09)	0.01 (−0.04, 0.07)	0.01 (−0.04, 0.06)	0.01 (−0.03, 0.06)	0.01 (−0.04, 0.05)
Obesity ^c,f^	0.19 * (0.11, 0.27)	0.19 * (0.11, 0.27)	0.17 * (0.03, 0.31)	0.18 * (0.03, 0.32)	0.00 (−0.10, 0.10)	0.00 (−0.11, 0.10)	0.04 (−0.06, 0.15)	0.04 (−0.07, 0.15)
Elevated WC ^d,g^	0.02 (−0.06, 0.11)	0.02 (−0.06, 0.11)	0.16 * (0.04, 0.28)	0.16 * (0.04, 0.28)	0.08 * (0.01, 0.14)	0.07 * (0.01, 0.14)	0.13 * (0.06, 0.20)	0.12 * (0.05, 0.19)
Insulin	0.01 * (0.01, 0.02)	0.01 * (0.01, 0.02)	0.01 * (0.01, 0.02)	0.01 * (0.01, 0.02)	0.01 * (0.01, 0.01)	0.01 * (0.01, 0.01)	0.01 * (0.00, 0.01)	0.01 * (0.00, 0.01)
Boys								
	12–14 years				15–17 years			
	TyG ^Φ^		TG/HDL ^δ^		TyG ^Φ^		TG/HDL ^δ^	
	Unadjusted	Adjusted	Unadjusted	Adjusted	Unadjusted	Adjusted	Unadjusted	Adjusted
Overweight ^b,f^	0.15 * (0.10, 0.21)	0.14 * (0.09, 0.19)	0.18 * (0.12, 0.25)	0.18 * (0.12, 0.24)	0.01 (−0.06, 0.09)	0.01 (−0.06, 0.09)	0.03 (−0.04, 0.10)	0.03 (−0.04, 0.10)
Obesity ^c,f^	0.21 * (0.12, 0.30)	0.19 * (0.10, 0.27)	0.33 * (0.22, 0.44)	0.33 * (0.22, 0.43)	−0.03 (−0.21, 0.14)	−0.03 (−0.19, 0.14)	−0.02 (−0.31, 0.26)	−0.01 (−0.29, 0.26)
Elevated WC ^d,g^	0.10 * (0.00, 0.20)	0.10 * (0.00, 0.21)	0.10 (−0.02, 0.22)	0.10 (−0.02, 0.22)	0.34 * (0.16, 0.53)	0.34 * (0.16, 0.51)	0.58 * (0.29, 0.87)	0.57 * (0.29, 0.85)
Insulin	0.01 * (0.01, 0.02)	0.02 * (0.01, 0.02)	0.01 * (0.01, 0.02)	0.01 * (0.01, 0.02)	0.02 * (0.02, 0.03)	0.02 * (0.02, 0.03)	0.02 * (0.01, 0.03)	0.02 * (0.01, 0.03)

^‡^ Adjusted for physical inactivity, smoking status, sedentary behavior, alcohol consumption, socioeconomic status, and Tanner stage; ^b^ Z-score > +1 and Z-score ≤ +2; ^c^ Z-score > +2; ^d^ ≥90th percentile for those aged 10 to <16 years old; ≥90 cm for males and ≥80 cm for females for those aged 16 years or older; ^f^ reference category: normal nutritional status; ^g^ reference category: normal waist circumference; ^Φ^ triglyceride/glucose index; ^δ^ triglyceride/high-density lipoprotein; * *p*-value ≤ 0.05.

**Table 3:** Unadjusted and adjusted ^‡^ fasting insulin equivalent (FIeq) for overweight, obesity, and elevated WC with insulin resistance markers and 95% confidence intervals stratified by sex and age in 37,815 adolescents included in the Study of Cardiovascular Risk Factors (ERICA, 2013–2014).

Girls								
	12–14 years				15–17 years			
	TyG ^Φ^		TG/HDL ^δ^		TyG ^Φ^		TG/HDL ^δ^	
	Unadjusted	Adjusted	Unadjusted	Adjusted	Unadjusted	Adjusted	Unadjusted	Adjusted
FIeq for overweight	0.72 (−3.49, 4.94)	1.33 (−2.03, 4.70)	2.35 (−3.14, 7.83)	2.70 (−2.34, 7.74)	1.49 (−4.34, 7.31)	1.13 (−4.48, 6.74)	1.38 (−3.99, 6.74)	0.99 (−4.32, 6.31)
FIeq for obesity	13.82 * (5.89, 21.76)	13.94 * (5.70, 22.19)	12.86 (−3.66, 29.39)	13.12 (−4.35, 30.60)	−0.20 (−10.99, 10.59)	−0.41 (−11.18, 10.36)	5.15 * (−7.38, 17.68)	4.76 (−7.94, 17.46)
FIeq for elevated WC	1.87 (−6.04, 9.78)	1.84 (−5.73, 9.43)	11.62 * (1.35, 21.90)	11.62 * (1.35, 21.89)	7.70 (−1.66, 17.06)	7.15 (−1.68, 15.97)	14.75 * (0.92, 28.58)	14.37 * (0.64, 28.11)
Boys								
	12–14 years				15–17 years			
	TyG ^Φ^		TG/HDL ^δ^		TyG ^Φ^		TG/HDL ^δ^	
	Unadjusted	Adjusted	Unadjusted	Adjusted	Unadjusted	Adjusted	Unadjusted	Adjusted
FIeq for overweight	10.32 * (5.42, 15.21)	8.98 * (4.89, 13.07)	12.42 * (5.54, 19.31)	12.44 * (6.22, 18.67)	0.64 (−3.45, 4.73)	0.66 (−3.68, 4.99)	1.62 (−2.54, 5.79)	1.74 (−2.71, 6.19)
FIeq for obesity	14.05 * (6.09, 22.00)	12.42 * (5.21, 19.63)	22.29 * (8.94, 35.64)	22.70 * (9.20, 36.19)	−1.32 (−11.38, 8.74)	−1.08 (−10.76, 8.60)	−0.99 (−18.60, 16.62)	−0.55 (−17.61, 16.51)
FIeq for elevated WC	6.89 (−2.17, 15.95)	6.33 (−2.58, 15.84)	6.78 (−4.07, 17.62)	6.91 (−4.35, 18.18)	15.71 * (3.25, 28.17)	15.70 * (3.70, 27.71)	29.28 * (7.33, 51.23)	29.26 * (7.87, 50.66)

^‡^ Adjusted for physical inactivity, smoking status, sedentary behavior, alcohol consumption, socioeconomic status, and Tanner stage; ^Φ^ triglyceride/glucose index; ^δ^ triglyceride/high-density lipoprotein; * *p*-value ≤ 0.05.

## Data Availability

The data presented in this study are available upon request from the corresponding author. The data are not publicly available due to the difficulty in identifying all the different databases and identifying the variable names. All manuals are in Portuguese.

## Supplementary Materials

The following supporting information can be downloaded at https://www.mdpi.com/article/10.3390/nu14173487/s1: Figure S1: Adjusted ** fasting insulin equivalents for overweight, obesity, and elevated waist circumference for insulin resistance markers (75th percentile value as the cut-off value) according to age and sex in The Study of Cardiovascular Risk Factors (2013–2014). ** Adjusted for physical inactivity, smoking status, sedentary behavior, alcohol consumption, socioeconomic status, and Tanner stage; ^1^ triglyceride/glucose index; ^2^ triglyceride/high-density lipoprotein; * *p*-value ≤ 0.05.
