# Supplementary material for: Association between Obesity, Overweight, Elevated Waist Circumference, and Insulin Resistance Markers among Brazilian Adolescent Students
Source: Nutrients. 2022 Aug 24;14(17):3487. doi: 10.3390/nu14173487 (PMC9458155; doi:10.3390/nu14173487)
Supplement: Supplementary file 1 [file nutrients-14-03487-s001.zip › nutrients-1847488-supplementary.pdf]

Supplementary Figure S1. Adjusted ** fasting insulin equivalents for overweight, obesity and elevated waist circumference for insulin resistance markers (75th percentile value as the cut-off value) according to age and sex in The Study of Cardiovascular Risk Factors (2013-2014)

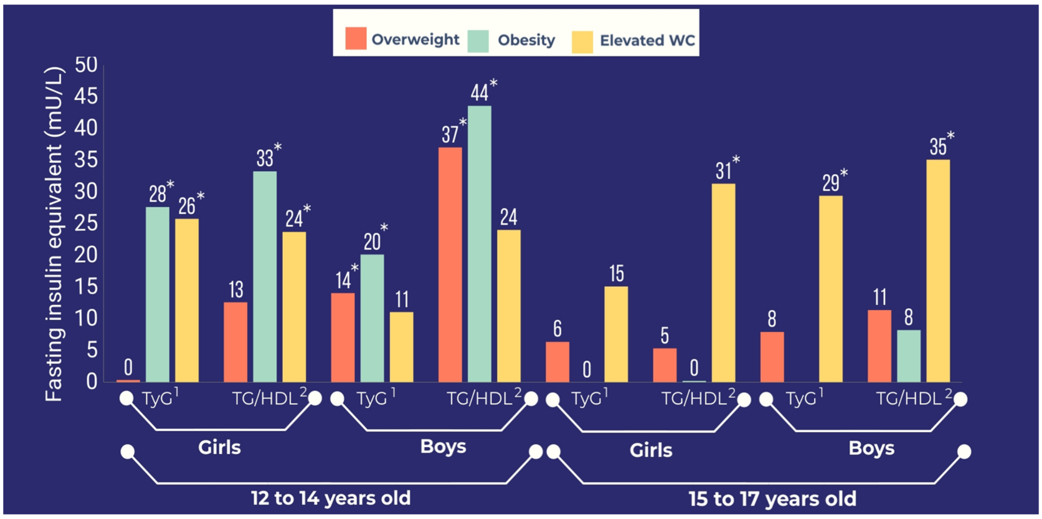

** Physical inactivity, smoking status, sedentary behavior, alcohol consumption, socioeconomic status and Tanner stage
[1] Triglyceride/glucose index
[2] Triglyceride/high-density lipoprotein
* p-value ≤ 0.05
